# Supplementary figures and images for: Host factor Rab11a is critical for efficient assembly of influenza A virus genomic segments
Source: PLoS Pathog. 2021 May 10;17(5):e1009517. doi: 10.1371/journal.ppat.1009517 (PMC8136845; doi:10.1371/journal.ppat.1009517)

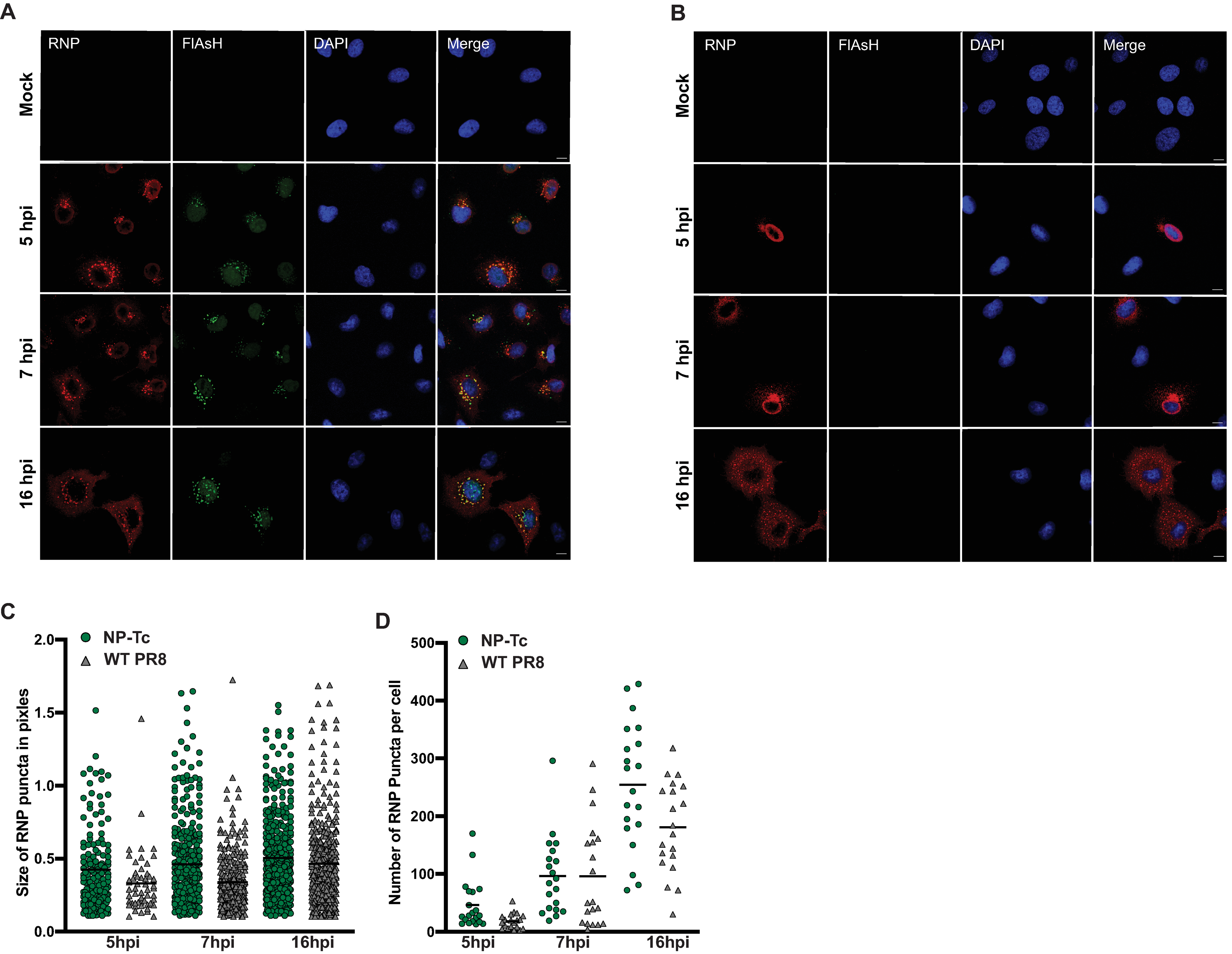

Supplement: S1 Fig — (A-B) A549 cells were infected with WT PR8 or NP-Tc virus at MOI = 1 for the indicated time points and stained with FlAsH dye and an anti-RNP antibody. (C) Comparison of RNP puncta sizes between NP-Tc and WT PR8 viruses. (D) Comparison of number of RNP puncta in control A549 cells infected with NP-Tc and WT PR8 viruses. Scale bar = 10 μm. Data are representative of at least two independent experiments. (TIF) [file ppat.1009517.s001.tif]

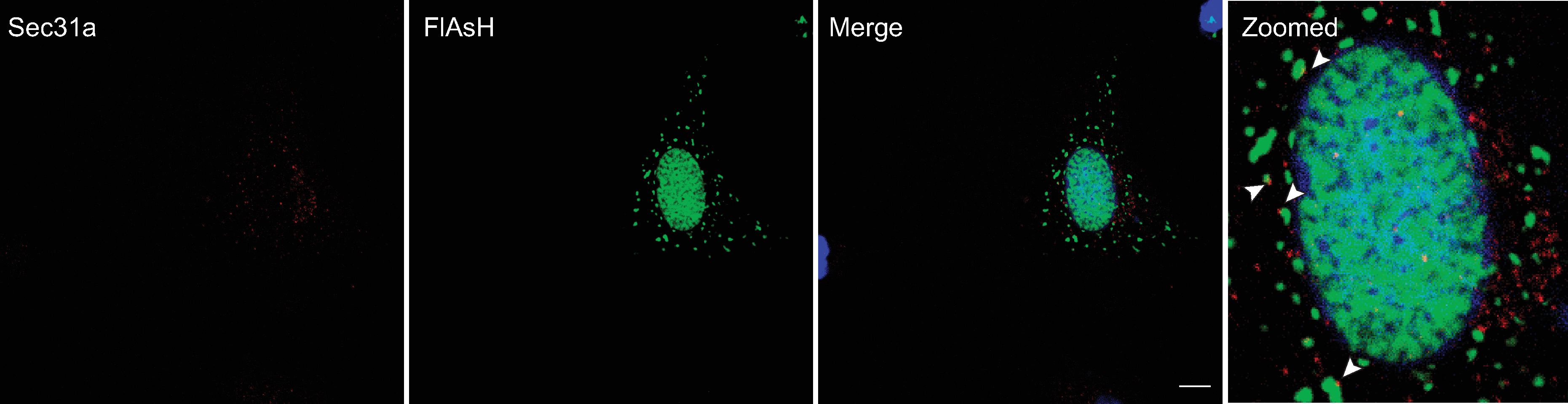

Supplement: S2 Fig — Control A549 cells were infected with NP-Tc virus and at 16hpi, cells were stained with FlAsH dye and a Sec31a antibody. RNP puncta with Sec31a in the periphery are indicated by arrows in the zoomed image. (TIF) [file ppat.1009517.s002.tif]

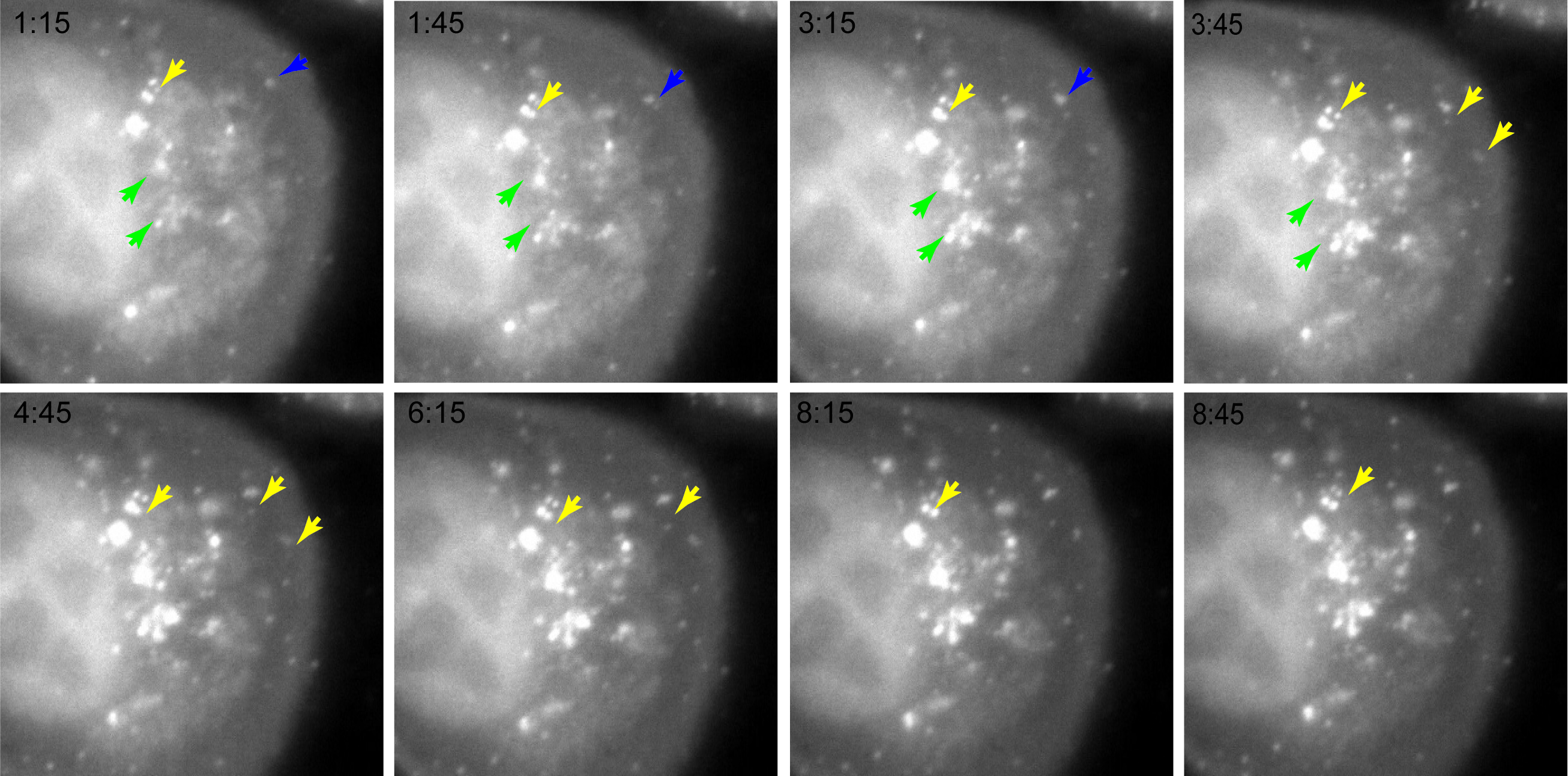

Supplement: S3 Fig — Selected frames from S1 Movie are shown with time (min:sec). Arrows indicate different events: RNP accumulation (green), puncta splitting (yellow) or coalescing (blue). (TIF) [file ppat.1009517.s003.tif]

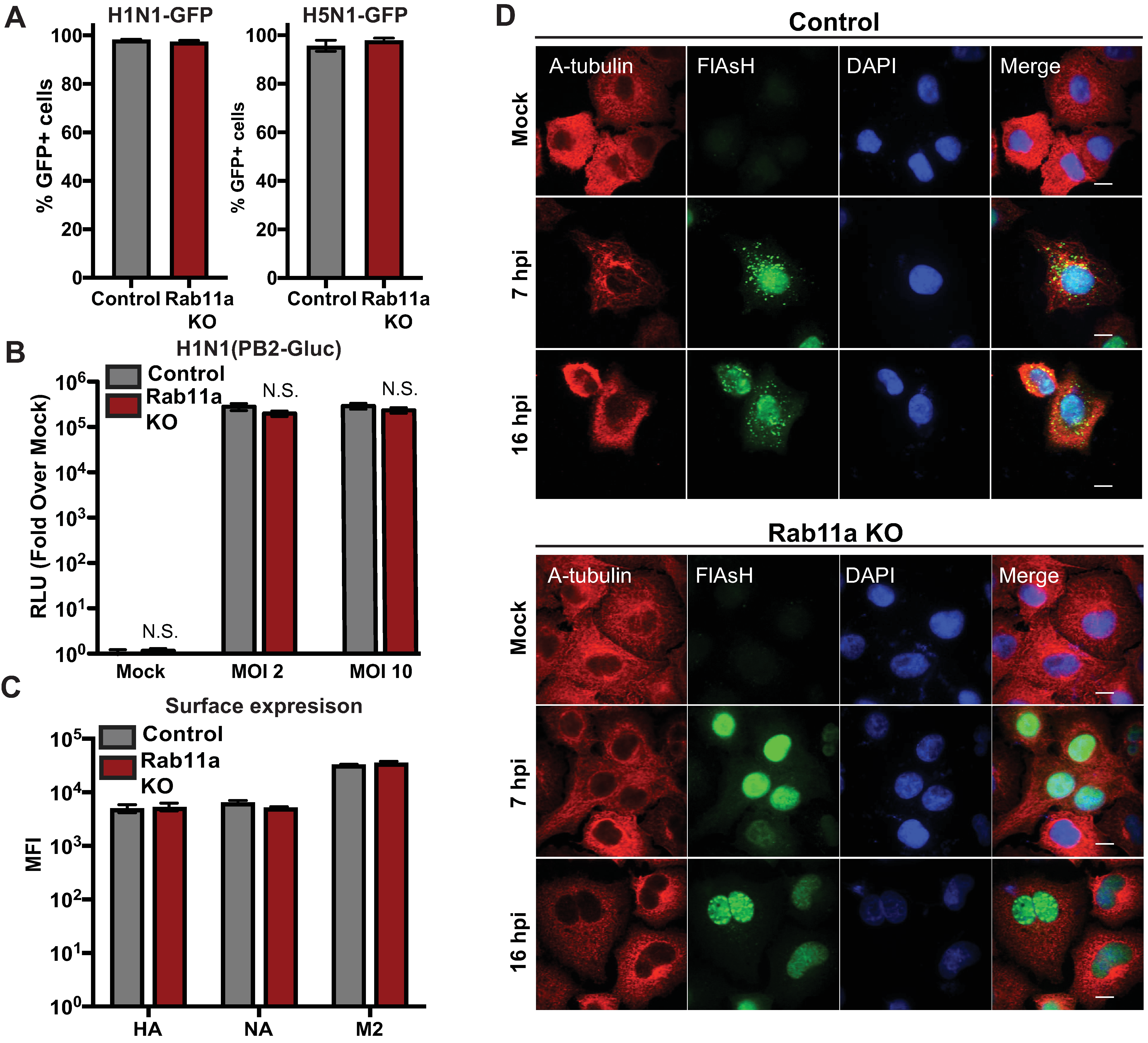

Supplement: S4 Fig — (A-B) Rab11a KO cells show defects at a post replication step. (A) Control A549 and Rab11a KO cells were infected with H1N1-GFP (PR8; MOI = 3) and H5N1-GFP (VN04; MOI = 1) and GFP expression was assessed at 16hpi by flow cytometry. (B) Control A549 and Rab11a KO cells were infected with H1N1 (PB2-Gluc; PR8) at MOI = 2 or 10 and incubated in media without TPCK trypsin to restrict infection to a single round. At 16hpi, luciferase activity in cell lysates was measured and is shown relative to mock lysates. Values are shown as relative light units (RLU). (C) Surface expression of viral proteins are not affected in Rab11a KO cells. Control A549 and Rab11a KO cells were infected with WT PR8 at MOI = 1 and surface expression of viral HA, NA and M2 were measured by flow cytometry. (D) Rab11a KO cells lack large FlAsH puncta. Control A549 and Rab11a KO cells were infected with NP-Tc virus at MOI = 1 for the indicated times and FlAsH staining as well as a-tubulin staining were performed. Scale bar = 10 μm. Data shown is a representative of two independent experiments performed with three biological replicates. (TIF) [file ppat.1009517.s004.tif]

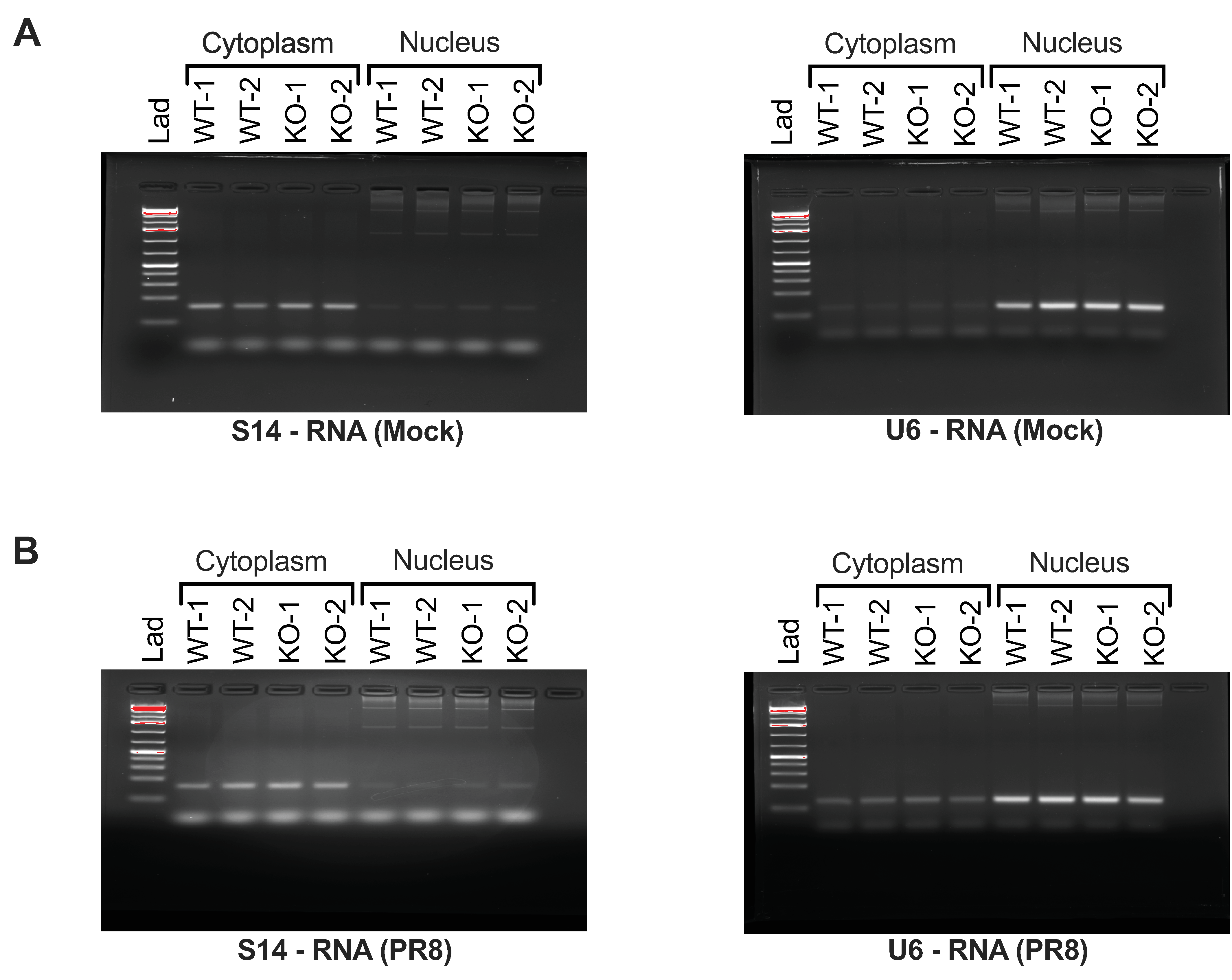

Supplement: S5 Fig — Control A549 and Rab11a KO cells were either mock (A) or WT PR8 infected (B), and at 7hpi, RNA from nuclear and cytoplasmic fractions were isolated. The quality of the fractions was assessed by semi-quantitative RT-PCR using S14 and U6 specific primers for 18 and 24 cycles, respectively. Data shown is a representative of two independent experiments performed with two biological replicates. (TIF) [file ppat.1009517.s005.tif]

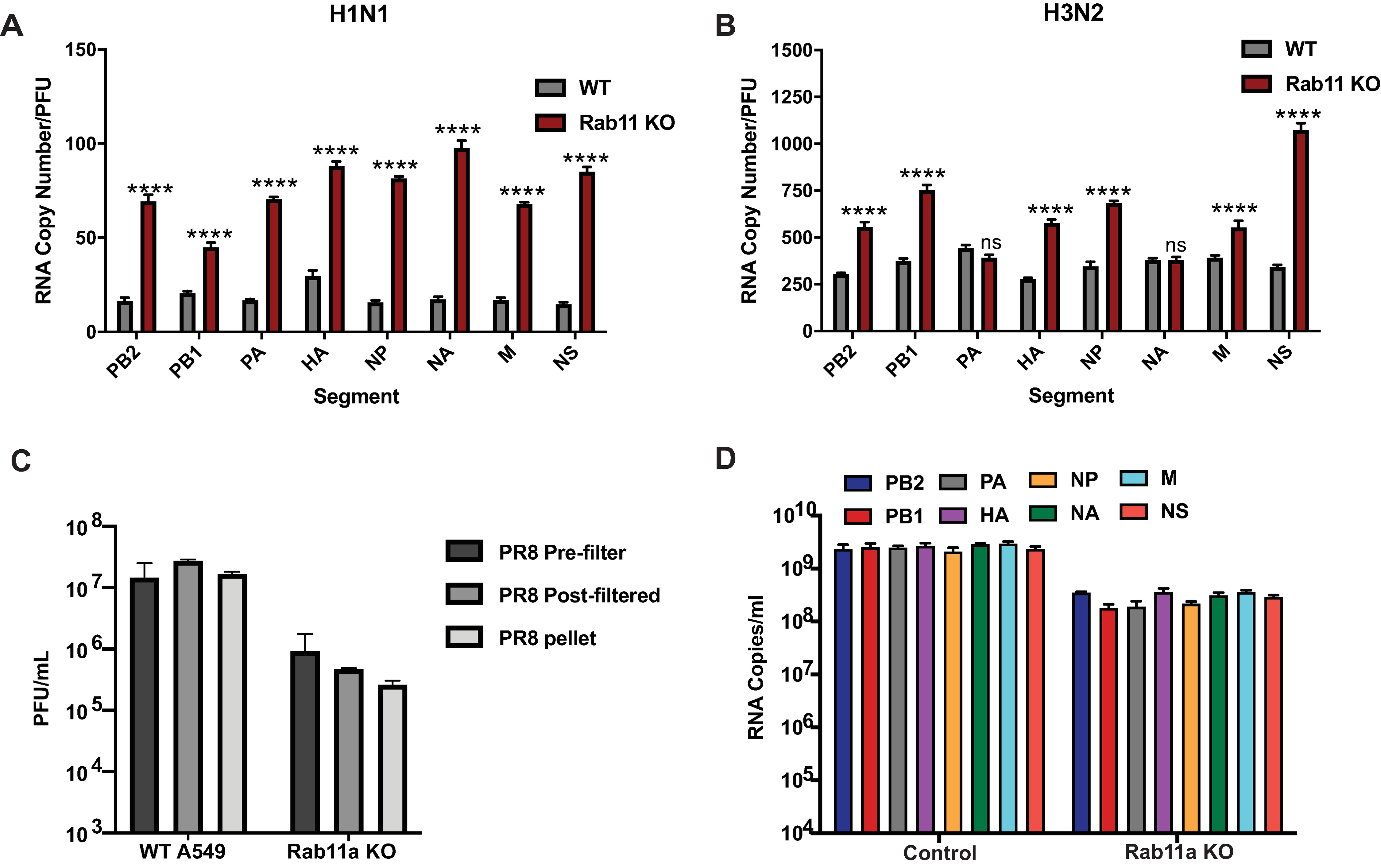

Supplement: S6 Fig — Rab11a KO cells show increased production of defective virions. (A-B) Control A549 and Rab11a KO cells were infected with H1N1(PR8) or H3N2 (Pan99) for 48 hours, and supernatants were tittered by plaque assay and subjected to viral RNA extraction. Viral RNAs were reverse transcribed and individual segment copy numbers were quantified by digital droplet PCR. The ratios of viral RNA copy number to PFU are shown for all eight segments of H1N1 and H3N2 viruses. (C) Viral titers at different stages of virion purification. Control A549 and Rab11a KO cells were infected with WT PR8 at MOI = 0.01 and at 72hpi, supernatants were collected and clarified of debris by low speed centrifugation. Subsequently, supernatants were filtered through a 0.45uM filter and pelleted on a 25% sucrose cushion. (D) Comparison of vRNA copy numbers in virions isolated from control A549 and Rab11a KO cells. * p-value < 0.05; ** p-value < 0.01; *** p-value < 0.001; N.S., non-significant. Data shown is a representative of two independent experiments performed with three biological replicates. (TIF) [file ppat.1009517.s006.tif]
