# Supplementary material for: Host factor Rab11a is critical for efficient assembly of influenza A virus genomic segments
Source: PLoS Pathog. 2021 May 10;17(5):e1009517. doi: 10.1371/journal.ppat.1009517 (PMC8136845; doi:10.1371/journal.ppat.1009517)
Supplement: Attachment — Submitted filename: Reviewer response-Revision.docx [file ppat.1009517.s008.docx]

Reviewer's Responses to Questions

Reviewer #1: OVERVIEW:

The authors investigate how the segmented influenza A virus genome is assembled in infected cells. This is important as it is the mechanism underlying the formation of epidemic and pandemic influenza A virus (IAV) genomes. This manuscript uses two new systems - a new virus where vRNPs are tagged in productive infections and Rab11a KO cells - and validates the system revisiting many aspects of how Rab11a contributes to IAV infection. It also adds some more evidence to the recently proposed role of Rab11-vRNP puncta being sites dedicated to IAV genome assembly. However, in the present state this reviewer is not able to recommend the manuscript for publication as it needs substantial work in improving the quality of the data, in the justification of using the new virus, in the characterization of the Rab11 KO cell line and in the validation of the work.

DETAILLED ASSESSMENT:

In the present manuscript the authors investigated the role of the recurrently characterized host protein, Rab11a, in IAV lifecycle. It has previously been shown that IAV vRNPs, after nuclear export, accumulate 1st around the MTOC to then associate with Rab11a or the ER, to then become dispersed in distinct puncta in the cytoplasm(Amorim et al., 2011; Avilov, Moisy, Munier, et al., 2012; Avilov, Moisy, Naffakh, & Cusack, 2012; Eisfeld, Kawakami, Watanabe, Neumann, & Kawaoka, 2011; Momose et al., 2011).

These puncta were shown to lead to the concentration of the 8-types of vRNPs in a Rab11 dependent manner (Chou et al., 2013; Lakdawala et al., 2014). The authors corroborate these findings in a new system, that although tagging vRNPs in productive infections as done before (Avilov, Moisy, Naffakh, et al., 2012; Bhagwat, Le Sage, & Lakdawala, 2018), tags a different constituent of vRNPs - NP.

In addition, authors use Rab11a KO cell lines rather than siRNA treated cells to

assess the role of Rab11a in IAV infection. More, similarly to what was demonstrated in (Alenquer et al., 2019), the authors propose that Rab11a serves to form sites dedicated for the assembly of influenza A virus genome. So, in essence the manuscript is not new. Nevertheless, the manuscript increases the list of IAV subtypes that depend on Rab11a for replication and makes an interesting and significant contribution by comparing the RNA copy number: PFU ratio of viruses in WT and Rab11a KO cells. This serves to confirm, in an elegant way, that the Rab11 sites formed in the cytosol are key for assembling viral genomes. In itself, this is sufficiently interesting for publication. However, the data presents needs better controls and validation as detailed below.

We would like to thank this reviewer for their insightful comments and suggestions, and for acknowledging the importance of our work in demonstrating the role of Rab11a in genome assembly. In the revised manuscript, we have provided additional evidence and controls that further support our findings. We addressed the majority of the concerns raised by this reviewer and included our responses below. We would like to point out that our study provides new evidence that loss of Rab11a results in increased numbers of defective particles.

Reviewer #2: In this study by Han et al., the authors investigated the function of host factor Rab11a on the efficient packaging of influenza A virus genome segments. The authors generated the recombinant virus carrying a tetracysteine tag in the NP gene (NP-Tc) which allows to analyze the dynamics of NP localization. By using the NP-Tc virus, the authors found that vRNPs were exported to the MTOC at early time points and then vRNPs were transported to the plasma membrane by interacting with Rab11a and microtubule networks.  Since disruption of microtubule networks by nocodazole failed to inhibit the inter-segment interaction of distinct vRNA, the authors hypothesized that Rab11a is a key factor regulating the selective packaging. By using Rab11a KO A549 cells, authors found that the assembly of vRNP puncta was significantly inhibited in Rab11a deficient cells. It was also revealed that the ratio of infectious virus per HA unit was significantly reduced and the levels of all

8 vRNA normalized with PFU number increased by Rab11a KO.  These results suggest that Rab11a is essential for the efficient packaging of vRNP segments.

However, there are several concerns to be addressed as follows. There is no evidence that the vRNP transport of NP-Tc virus is identical to the wild-type, although the data and description showed novel findings of the vRNP dynamics using NP-Tc virus. In addition, the authors have to address the contradictory results between the strong reduction in viral titer and the mild inhibition in viral replication steps. The method to examine RNA copy/PFU is not technically appropriate unless the purification of viral particles.

We have now included data comparing RNP trafficking during WT PR8 and NP-Tc virus infections (Supplementary Fig S1). These results demonstrate that RNP trafficking is similar for both viruses. As expected, due to the decreased replication of NP-Tc virus, the numbers of RNP puncta are lower in NP-Tc infected A549 cells as compared to WT PR8 infected cells.

In the mini-genome assay, luciferase reporter (NP-Luc-NP vRNA) expression is driven by viral polymerase components expressed from a chicken β-actin promoter. However, in the context of NP-Tc virus infection, virally encoded NP-Tc drives viral replication including NP expression. As such, a modest decrease in NP-Tc function can have a cascading effect on overall viral replication.

Our studies strongly indicate that the defects in Rab11a KOs occur at a post entry step. We have included data from single cycle high MOI infection experiments in which we observed an ~10-fold decrease in viral titers. However, in a multicycle low MOI experiments, there is a cumulative effect on replication, resulting in a 2-3 log decrease in replication.

In the revised manuscript, we have performed sucrose purification of virions and determined RNA copy numbers. As before, we observed higher vRNA copy/PFU ratio for virions purified from Rab11a KOs as compared to control A549 cells, demonstrating that Rab11a KOs produce higher numbers of defective particles (Fig 5).

Reviewer #3: This study by Han et al. documents how the host GTPase Rab11 is involved in the trafficking of the influenza virus segmented genome (in the form of viral ribonucleoproteins or vRNPs) across the cytoplasm of infected cells. The experimental approach is innovative in two respect: i) vRNPs were visualized by using a genetically engineered reporter virus that expresses a tetracysteine-tagged nucleoprotein, whereas previous studies relied on indirect immunofluorescence or on reporter viruses expressing a GFP or mCherry-tagged polymerase; and ii) the CRISPR/Cas9 method was used to engineer Rab11A knock-out cells, whereas previous studies relied on siRNA-mediated Rab11A knock-down. These methodogical advances will be of interest for readers in the field, especially as the reporter virus expressing a tetracysteine-tagged nucleoprotein might potentially be used for live imaging. In the present paper however, it was exclusively used to visualise vRNPs in fixed cells.

In general the study is well-conducted, except for a cell fractionation experiment whose design should be improved. Overall the data confirm previously published evidence that Rab11 is involved in vRNP transport. They are also compatible with the previously proposed model that the transport and the assembly of the viral genome are intimately related, and Rab11 is involved in both processes.

Although the authors claim that they further show that Rab11A+ vesicles “enable efficient packaging through specific vRNP:vRNP interactions”, the experimental data actually do not  demonstrate that the viral particles produced from Rab11A knock-out cells are presenting packaging defects. Unless new data are produced to support their claims, the conclusions and the model proposed by the authors should be revised, as well as the title of the manuscript.

We thank the reviewer for encouraging comments and suggestions. In the revised manuscript, we have determined the vRNA copy numbers for cytoplasmic and nuclear fractions from Rab11a KOs and control A549 cells. We have also validated the enrichment of cytoplasmic and nuclear fractions by semiquantitative PCR for cellular RNAs S14 and U6, respectively.

We agree with the reviewer’s comment that we do not directly demonstrate a packaging defect in virions produced from Rab11a KO cells. According to the reviewer’s suggestion, we have now modified the title to “Host Factor Rab11a is Critical for Efficient Assembly of Influenza A Virus Genomic Segments”.

--------------------

Major Issues: Key Experiments Required for Acceptance

Reviewer #1: 1)	The real advantage of using this new virus is that it allows evaluating vRNP progression using live cell systems. However, the authors have not done so. As the virus is attenuated, and as there is no real advantage in using the NP-Tc virus, authors should use wild-type viruses instead.

We have now included live imaging studies with NP-Tc virus in A549 cells expressing mCherry-Rab11a. We observed co-localization and movement of mCherry-Rab11a and NP-Tc. This recapitulates previous findings that RNPs are trafficked on Rab11a positive vesicles. We were only able to perform imaging studies for a 30min time period due to photo-bleaching of NP-TC, which is a caveat of the tetra-cysteine system and thus, have acknowledged this in the revised manuscript.

2)	NP-Tc viruses could be used to compare vRNP progression in the Rab11KO cells with that observed in WT cells in live systems. If doing so, Figures 2-3 should be condensed into a single figure that serves to validate the NP-Tc virus. The data is important because it serves to explain that NP-Tc vRNPs behave like vRNPs of WT viruses, despite the 2-log deficiency in viral replication.

We have included live imaging studies in WT A549 cells. However, as Rab11a KO cells do not show any distinct puncta, we could not observe any changes in NP-Tc trafficking in Rab11a KOs.

In the revised manuscript, we have combined figures 2 and 3. We have also performed immunostaining with anti-RNP antibodies and compared vRNP progression during NP-Tc and WT PR8 virus infection in WT A549 cells (Fig S1). In these studies, we observed similar progression of vRNP transport between NP-Tc and WT PR8. As anticipated, the numbers of RNP puncta were lower during NP-Tc virus infection as compared to WT PR8 infection in WT A549 cells, which is likely due to decreased polymerase activity associated with NP-Tc.

3)	Authors should explore the underlying reason for NP-Tc being attenuated.

In our mini-genome assays, NP-GFP or RFP showed no activity and NP-Tc showed 70% activity relative to WT NP. Here, the expression of PB1, PB2, PA and NP are driven by a strong chicken b-actin promoter. However, in the context of NP-Tc virus infection, transcription and replication are driven through virally produced components. As such, the reduction in NP-Tc activity has a cascading effect on viral replication.

4)	The other problem that I see with the data presented, is that vRNPs seem to be retained in the nucleus in the Rab11 KO cell lines, rather than being dispersed throughout the cytosol as claimed. This is notorious in the FIAsH, the PB2 vRNA and NP vRNA in Figure 5A, although in Figure 5C NP vRNA and PB2 vRNA less so. Please comment and see also point 5.5)	The authors try to show that there are no differences in the cytosolic amounts of vRNA in WT and Rab11KO cells (Figure 5F) but do not provide sufficient detail on the methodology for reviewers to be able to assess the quality of the data. For example, how have authors calculated total RNA levels? The values presented are relative levels (delta delta CT or "log fold-change") and therefore authors are not able to sum RNAcyt+RNAnucl. This is especially evident because RNA levels are normalized in relation to two different host mRNAs - tubulin and U6 RNA. The data is therefore not correct and copy number of the vRNA, rather than relative values should be used in this conditions.

We agree with this reviewer’s comments and included additional data to address these questions. To demonstrate that vRNPs are dispersed in the cytoplasm of Rab11a KOs, we performed immunofluorescence staining for vRNPs using an in-house made RNP antibody. RNP antibody staining showed small and dispersed signal for RNPs in the cytoplasm of Rab11a KO, supporting our model that vRNPs are dispersed in these cells (Fig 4C). We have included quantification for the sizes of RNP puncta observed in Rab11a KOs, which were significantly smaller than the RNP puncta observed in control A549 cells (Fig 4E). As suggested by this reviewer, we have now determined the vRNA segment copy numbers in the nuclear vs cytoplasmic fractions. Enrichment for nuclear and cytoplasmic fractions was also verified by semiquantitative RT-PCRs for U6 and S14 RNAs. In these studies, we did not observe significant differences in the vRNA copy numbers in the nuclear and cytoplasmic fractions, validating our findings that there are no defects vRNPs nuclear export in Rab11a KO cells (Fig 4F).

6)	Also in Figure 5F, authors should show controls for the purity of nuclear and cytoplasmic fractions. Furthermore, accordingly to (Kawakami et al., 2011), in conventional real time RT-PCR, the use of segment specific primers, leads to substantial background amplification of viral mRNA and cRNA. While the estimated amount of cRNA is very small compared to vRNA (2 logs difference), the mRNA levels are not, and could account for part of the amplification observed. Please comment.

As the reviewer points out, the major issue with conventional PCR is differentiating positive sense cRNA vs mRNA, but not with the negative sense vRNA copy number determination. We would like to clarify that in panel 5F of the previous submission only vRNA was determined. We have validated our vRNA strand-specific RT reaction with a universal primer that is specifically complementary to the 3’ end of the vRNA segments, and did not see any non-specific amplification.

7)	It is interesting that the authors comment on the manuscript of (de Castro Martin et al., 2017) but rather disappointing that they did not test whether newly exported vRNPs from the nucleus attached to ER membranes. This is something that should be done by determining if vRNPs co-localize with the ER in the Rab11KO cell lines over a course of infection.

To address this question, we performed Sec31a staining during NP-Tc infection. As previously reported by Alenquer et al., 2019, we observed association of Sec31 in the periphery of some of the FlAsH puncta but not all. We have now included this information in the supplementary section (Fig S2).

8) Many papers have commented on vRNP-Rab11a transport along microtubules (Avilov, Moisy, Munier, et al., 2012; Avilov, Moisy, Naffakh, et al., 2012; Bhagwat et al., 2018; Bhagwat et al., 2020; Eisfeld et al., 2011; Kawaguchi, Asaka, Matsumoto, & Nagata, 2015; Kawaguchi, Hirohama, Harada, Osari, & Nagata, 2015; Kawaguchi, Matsumoto, & Nagata, 2012; Momose, Kikuchi, Komase, & Morikawa, 2007; Momose et al., 2011; Vale-Costa et al., 2016). Here, the authors limited the analyses of depolymerising microtubules on vRNP localization in WT cells, as others have done. However, they now have an excellent novel tool, which is the Rab11KO cell line and should assess the relevance of microtubules, of actin and of ER in this. Also, please include the following manuscript in your discussion (Bhagwat et al., 2020).

As per this reviewer’s suggestions, we compared virus replication of PR8 virus in Rab11a KOs and control A549 cells, and observed no difference in replication in the presence or absence of Nocodazole. In addition, we also performed Sec31 staining along with FlAsH in WT cells. In agreement with the previous report by Alenquer et al., we observed NP-Tc Sec31 staining near the periphery of some of the NP-Tc puncta but not all, confirming the previous findings that RNPs may transit through ERES.

We have now included Bhagwat et al. in the discussion section.

9)	Are Rab11KO cell lines competent in transferrin recycling? This should be tested because Rab11 is an essential gene. Furthermore, the growth rate of these cells should be analysed in comparison to WT cells in order to validate the diference in titers observed. Also, authors should assess the localization of Rab11b in infected cells.

We have assessed transferrin recycling and observed reduced transferrin uptake and recycling in Rab11a KO cells as reported by others (Weigert et al 2005) . This is now shown in Fig 3C.

Our study and others show that Rab11a KO cells are viable (Yu et al 2014, Zulkefli et al. 2019, Homma et al., 2019. We do not observe any growth defects in Rab11a KO cells. If any, Rab11a KO cells show ~10% faster growth rate than WT A549 cells.

10)	Figure 5G aims to show that virions that come out of Rab11 KO cell lines are less infectious. However, a better way to show this is to actually count virions by electron microscopy and negative staining.

Assessment of defective particles can be determined by different ways. We are confident that PFU/HAU ratio as well as PFU/RNA copy number ratio can provide similar information.

11)	That Rab11 serves as hubs for the congregation of vRNP complexes and enable efficient genome packaging through specific vRNP:vRNP interactions has been proposed before (Alenquer et al., 2019). Here, the authors provide additional evidence for this. Please include the manuscript and comment both findings in your discussion.

We have now included this reference in the manuscript.

12)	It is unclear if the authors forgot to include the statistics in graphs of figure 6, or if the results are not significant. If the latter, then the results and the interpretation should be that there are no differences in packaging in both cases and the paper should not be published. Please comment.

We have included statistical analysis for Fig 5. Instead of using viral supernatants, we have purified the virus particles on a sucrose cushion and performed vRNA copy number analysis. Here we observed significant differences in the copy numbers for segments PB2, HA, NA, M and NS.

Reviewer #2: 1)	Fig1-3: The authors generated the recombinant virus carrying NP-Tc showing co-localization of FIAsH signals with NP protein, vRNA, Rab11a, and tubulin and claimed that the trafficking of NP-Tc vRNP was identical to that of wild-type virus.  However, the figures did not provide such evidences because there is no comparison with wild-type virus (e.g. Fig 1A, 2A, 2B, and 3A). It is also required to explain about the nuclear accumulation of FIAsH signals (e.g. Fig 1A, 2A, 2B, and 3A). The NP protein is required for the nuclear export of vRNP by its interaction with CRM1 exportin (Elton et al., 2001, PMID11119609). The NP-Tc signals are accumulated in the nucleus even at the 9 hpi (e.g. Fig 1E, 1F, and 2A). Is the nuclear export of vRNP inhibited or delayed in cells infected with NP-Tc virus? In addition, some of the results about vRNP dynamics are not novel findings. For instance, the association of vRNP with MTOC (Fig3A), dispersed vRNP puncta in nocodazole-treated cells (Fig3B), and conserved association of vRNA with distinct vRNA segment in nocodazole condition (Fig3C and 3D) have been reported (Jo et al., 2010, PMID 20637889, Momose et al., 2007, PMID17905627, and Chou et al., 2013, PMID23671419).

Based on this reviewer’s comments, we have now included comparisons of vRNP progression between WT PR8 and NP-Tc virus. To address the issue of vRNP accumulation in the nucleus vs dispersion in the cytoplasm, we have performed several experiments. First, we have used RNP antibody staining to demonstrate the presence of vRNPs in the cytoplasm of Rab11a KO cells. Second, we have isolated cytoplasmic and nuclear fractions from PR8 infected WT and Rab11a KO A549 cells and determined vRNA copy numbers. We did not observe significant differences in the vRNA copy numbers between WT and Rab11a KO cells. Third, nocodazole experiments were included to demonstrate that the NP-Tc virus recapitulates WT vRNP dynamics. We believe our studies provide the important experimental evidence that Rab11a act as a hub for congregation of vRNPs and thus, promotes specific genome assembly.

2)	Fig4-6: In Fig 4B-D, the authors observed 3-4 logs lower viral titers of several IAV strains in Rab11a KO cells compared with that in control cells. The observed reduction is obviously stronger than that in siRab11a cells with 20-fold reduction. Although the author explained with a functional complementation possibly by Rab11b or Rab25 in Rab11a KD condition, however, there is lack of evidence that Rab11b and Rab25 are surely inhibited in this study. Besides, the authors concluded that a dispersed localization of vRNP in Rab11a KO cells (Fig 5C) may result in inefficient packaging of vRNP, and this is a main cause of reduction in viral replication. However, it seems not enough to explain the strong reduction in virus production only by the dispersed pattern of vRNP, because a similar vRNP localization is observed even in Rab11a KD cells (Amorim et al., 2011, PMID21307188). The Rab11a knockout may widely affect endosomal pathways, because Rab11a regulates not only a cargo

trafficking through the recycling endosomes but also a transport of post-Golgi vesicles or homeostasis of the endosomal-lysosomal pathway (Zulkefli et al., 2019, PMID30981667). Thus, to confirm a dependency of viral replication on Rab11a, the authors need to examine the following.

We agree with the reviewer’s comment that there is no evidence for Rab11b or Rab25 compensating for Rab11a. Hence, we have now removed this from the discussion.

Amorim et al. performed replication experiments in Rab11a KD A549 cells at a high MOI (MOI=5) and observed a 10-fold decrease in replication at 24hpi. Our experiments shown in Fig 3 (revised manuscript) were performed at a low MOI (0.01-0.001) and the observed 3-log decrease in viral titers was due to accumulative effects over multiple rounds of replication. We have now included high MOI experiments in Rab11a KOs for PR8 strain and VN04 strains. We also observed an ~10 fold decrease in replication in Rab11a KO cells as compared to control A549 cells.

---Is the transport of viral membrane proteins to the plasma membrane affected by Rab11a KO?

As per this reviewer’s suggestions, we have performed flow cytometry analysis for surface expression of viral HA, NA and M2 proteins, and observed similar mean fluorescence intensities for viral surface proteins between WT and Rab11a KO cells (S3C Fig).

---To show the infected cell ratio in control and Rab11a KO cells with same MOI of viruses.

To demonstrate equal susceptibility of WT and Rab11a KO cells, we have performed single cycle infections with PR8-GFP and VN04-GFP, and determined the relative % GFP expressing cells. We did not see any significant differences in the levels of GFP+ cells (S3A Fig). In addition, we have performed infections with a PR8 (PB2-GLuc) luciferase reporter virus and observed similar levels of luciferase expression in both WT and Rab11a KO cells (S3B Fig).

3)	Fig 5A and 5B: In the case of NP-Tc virus infection, vRNP signals predominantly localize in the nuclei while which localize throughout the cytoplasm in wild-type virus.

As FlAsh dye can bind to free NP as well as RNPs, the intense FlAsH staining seen in the nuclei could be in part due to FlAsH binding to free NP. In terms of vRNP staining via FISH, we observed slightly higher FISH signal in the nuclei of NP-Tc infected cells as compared to the nuclei of WT PR8 infected cells. This is likely due to higher replication of WT PR8 virus as compared to NP-Tc virus, resulting in higher accumulation of vRNPs in the cytoplasm. Consequently, our imaging analysis displays intense cytoplasmic signal in WT PR8 infected cells.

4)	Fig 5F: Why the level of cytoplasmic vRNA is more than total vRNA?

This was due to normalization with different controls (U6 and tubulin) for cytoplasmic and nuclear fractions. To address this issue, we have now performed digital droplet PCR (ddPCR) and determined the vRNA copy numbers in the nuclear and cytoplasmic fractions instead of normalization to U6 and tubulin RNA (Fig 5).

5)	Fig 6A-D: To exclude the possibility of vRNA contamination from dead cells, the authors have to examine the copy number of vRNA after the virion purification by ultracentrifugation.

As per the reviewer’s suggestion, we have performed analysis with sucrose purified virions and included this data in the revised manuscript (Fig 5). In sucrose purified virions, we also observed increased vRNA copy number/PFU and vRNA copy number/HAU ratios for virions purified from Rab11a KO supernatants as compared to virions purified from control A549s (Fig 5).

6)	It has been reported that influenza A virions mostly package eight vRNPs (Nakatsu et al., 2016, PMID2760575) and even a seven segments virus is able to package eight RNPs incorporating host ribosomal RNAs (Noda et al., 2018, PMID2930061). In Fig 6A-6D, the authors found the increased level of vRNA/PFU ratios of all eight vRNA segments in Rab11a KO cells, indicating that Rab11a KO results in the production of defective viral particles, possibly impaired incorporation of all vRNA segments in viral particles. However, the vRNP configuration in virions produced form Rab11a KO cells is still unclear, because there is a possibility that there are increased levels in empty virions or virions contain less than eight vRNPs. Thus, it is required to address whether Rab11a+ endosomes are required for general incorporation of vRNPs, selective packaging, or both.

This reviewer raises an important and interesting question for the influenza virology field. If there are more empty virions produced from Rab11a KO cells, one would expect to see lower vRNA/HAU ratio for virions produced in Rab11a KOs as compared to virions produced from WT A549 cells. However, in our studies, virions produced from Rab11a KO cells showed higher vRNA copy number/HAU ratio, suggesting that there are likely no defects in general incorporation of vRNAs (Fig 5). Also, as the reviewer points out, influenza virus incorporates ribosomal RNA in 7 segmented mutant viruses. We speculate that additional copies of the same segment may likely be incorporated to maintain the 7+1 configuration.

Reviewer #3: Figures 3C and 5A. Standard FISH analysis does not allow to accurately assess the degree of colocalization of the NP and PB2 vRNAs. Single-molecule FISH analysis would be more accurate. However even the conclusions drawn from single-molecule FISH are limited by the fact that its 100nm resolution is not sufficient to conclude that co-localizing vRNPs represent physically associated vRNPs.

In Figure 5A-B, the authors assume that large NP-Tc puncta are “supramolecular vRNP structures associating vRNPs corresponding to different viral genomic segments”, however this remains very speculative as i) their data do not demonstrate the presence of such supramolecular structures associating different types of vRNPs, and ii) a previous study (Alenquer et al, 2019) has shown that large NP-positive, Rab11-positive puncta can be formed in cells that express a single type of vRNP.

We agree with the reviewer’s comment that standard FISH does not accurately reflect co-localization. Considering the fact that large distinct punta in the cytoplasm showed the presence of 3 different vRNP segments, it is highly likely that these are vRNP bundles as suggested by others and us.

Figure 5F. The design of the cell fractionation experiment should be improved by using a strand-specific RT-qPCR protocol, and by controlling the purity of the nuclear and the cytoplasmic fractions. In the present state, no conclusions can be drawn from this experiment.

In the revised manuscript, we have shown enrichment for nuclear and cytoplasmic fractions by semiquantitative RT-PCR using U6 and S14 RNAs, respectively (Supplementary Fig X). As suggested by this reviewer, we have also performed negative sense vRNA specific RT reactions using a universal influenza virus primer that binds to the complementary sequence in negative sense RNA and have determined the copy numbers for PB2, NP and M vRNAs using segment specific primers.

Figure 6 and 7. The 2-fold increase of the RNA copy number to PFU/ratio in the Rab11-KO cells is a very minor difference compared to the 3-log decrease in infectious viral titers. If Rab11 was critical for the assembly of sets of 8 distinct vRNPs as claimed in the title and abstract of the manuscript, and if random assembly of vRNPs occurred in Rab11-KO cells as suggested in Figure 7, one would expect a much stronger increase in the proportion of non-infectious viral particles. The 2-fold increase in non-infectious viral particles could possibly be unrelated to vRNP assembly and packaging. For instance, the defect in Rab11 could possibly alter the composition of the plasma membrane and therefore the composition of the viral envelope. The data presented in the manuscript confirm that in the absence of Rab11 the overall efficiency of vRNP transport and assembly is strongly decreased, but they do not provide any demonstration that the viral particles produced from Rab11-KO

cells are presenting packaging defects. Single viral particle analysis would be required to assess this point.

We agree with this reviewer’s point that we do not directly demonstrate defects in vRNP packaging. Accordingly, we have corrected the abstract and title to indicate that the lowered viral titers in Rab11a KOs are likely due to defects in vRNP assembly or bundling.

We kindly disagree with the reviewer’s assessment that there is only a two-fold increase in defective particles due to the loss of Rab11a. Based on our analysis of PFU/HAU ratio (infectious particles/total particles), we observed an ~20 fold increase in defective particles in Rab11a KO cells as compared to control A549 cells (Fig 4G). In the revised manuscript, we included the results of high MOI infection replication experiments (single cycle). In these experiments, we observed an ~10-fold decrease in viral titers, which is in agreement with previous siRNA KD studies. However, in low MOI multi-cycle replication experiments, we observed a >3 log decrease in viral titers due to the cumulative effects of loss of Rab11a.

While Rab11a is important for large vRNP assembly/bundle formation, smaller vRNP bundles are still formed through vRNA:vRNA interactions in Rab11a KO cells. We believe these interactions also contribute to production of infectious particles. We also performed antibody staining for viral HA, NA and M2 proteins and observed no differences in the mean fluorescence intensity, suggesting that expression of viral surface proteins was unaffected in Rab11a KO cells. Moreover, we have performed single cycle infections with H1N1-GFP and H5N1-GFP viruses and observed no differences in susceptibility between Rab11a KOs and control A549 cells.

--------------------

<b>Part III – Minor Issues: Editorial and Data Presentation

Reviewer #1: 13)	Summary. line 18: not all influenza viruses have 8 segments. Please correct

We have corrected this.

14)	Line 19, the word are at the end should be is

We have corrected this.

15)	Page 4 line 77 – include references after “no significant co-localization between different vRNP segments” because this has been done already – (Chou et al., 2013; Lakdawala et al., 2014).

We have included these references.

16)	Figure 1 - Include the NP-200 virus in the growth curve and discuss.

We have included NP200 virus in the growth curve.

17)	Join figure 2 and 3 to validate the virus.

We have merged figures 2 and 3 together. Some of the data has been included in the supplementary section.

18)	Page 11 – line 215 -217 – duplicated in text, please delete.

We have revised this section in the manuscript.

19)	Discussion – line 282 – the tag is not fluorescent – please correct.

We have removed the term fluorescent.

20)	Lines 145, 160 and 292. Please explain why you wrote polarized.

The term polarized was used to emphasize the movement of RNPs towards the plasma membrane away from the MTOC. We have removed the term polarized to avoid confusion with polarized epithelial cells.

21)	In the Rab11KO cells, do vRNPs co-localize with the ER? This is very important, as it will contribute to elucidate the role of the ER in vRNP transport.

We have now included Sec31a staining along with FlAsH staining. As reported by Alenquer et al., Sec31a puncta were present in the periphery of a few vRNP FlAsH puncta and we have included this data in Fig SX.

22)	Figure 4 lacks statistical analyses in H1N1; H5N1; H3N2 and H7N7.

We have now included statistical analysis in this figure (Fig 3).

23)	Figure 5 B and C FIAsH staining is restricted to the nucleus in Rab11 KO cells. In Figure 5C the two vRNPs tested are also restricted to the nucleus. Please comment.

Based on FIAsH staining, we observed intense FlAsH staining in the nucleus of Rab11a KOs and this is likely due to FlAsH binding to free NP in the nucleus. In addition, the lack of intense cytoplasmic RNP puncta in Rab11a KO cells accentuates the nuclear signal. We have now included RNP specific antibody staining along with FlAsH staining, which shows the presence of small RNP puncta in the cytoplasm (Fig C).

24)	Figure 5C is could either show more cells or if showing only 1-2 cells could be zoomed in.

We have now included the zoomed image of the cells.

25)	Please revise statistics of Fig5D for VSV.

There is a modest 2 fold decrease in VSV titers in Rab11a KOs and thus, statistical analysis shows significance.

26)	Do budding virions still contain 8-vRNPs? This could be tested by transmission electron microscopy.

Based on Noda et al., 2018, it is likely that virions from Rab11a KOs will incorporate 8 vRNPs to maintain a 7+1 configuration. In our analysis of sucrose purified virions from Rab11a KOs, we did not see a difference in the copy numbers of individual vRNP segments, suggesting no defects in the incorporation of specific vRNPs. We speculate that some of the virions may not contain 8 individual segments, likely contributing to the production of defective particles.

Reviewer #2: (No Response)

Reviewer #3: Figure 2B. The authors mention an increase in size of the NP-Tc puncta over time, which is not obvious on the figure and could possibly be quantified as in Figure 5D.

We have moved these images to the supplementary section and have included the quantification of puncta sizes.

Figure 3A. The image shown as representative of the 5 hpi time point is quite different from the 5 hpi image shown in Fig 2B. Could the authors comment on the synchronicity of infection and the cell to cell heterogeneity ?

Despite synchronizing the infections on ice, we saw some heterogeneity in RNP trafficking between experiments. Between 3-5 hpi, we saw accumulation of RNPs near the MTOC. The representative image from 3A (Now Fig 2B) is from a 5hr time point.

Figure 4. Replication of the VSV is not altered in Rab11 KO cells, which provides an indirect control of cell viability. Did the authors notice any apparent change in cell morphology or behaviour upon Rab11 knock-out ? Adding this information might be useful to the readers.

We passaged Rab11a KO cells over 25 passages and did not observe any noticeable changes in cell morphology. If any, Rab11a KOs show a modest increase in growth as compared to parental WT A549 cells. We have now included this information in the manuscript (LN).

--------------------
